# Association of human blood metabolites with rheumatoid arthritis: A Mendelian randomization study

**DOI:** 10.1097/MD.0000000000041666

**Published:** 2025-03-07

**Authors:** DeZhi Yan, Di Luo, Wei Yan, Hao Liu, JinSong Li

**Affiliations:** a The First Clinical College of Medicine, Shandong University of Traditional Chinese Medicine, Jinan, Shandong Province, China; b Department of Orthopedic Joints, Affiliated Hospital of Shandong University of Traditional Chinese Medicine, Jinan, Shandong Province, China.

**Keywords:** blood metabolites, causality, Mendelian randomization, rheumatoid arthritis

## Abstract

Rheumatoid arthritis (RA) is a common autoimmune disease, but its specific biological mechanisms, especially the link between metabolites and RA, have not been conclusively hypothesized. This study used a Mendelian randomization (MR) analysis approach to assess the association between 1400 human blood metabolites and risk of RA. Pooled statistics of 1400 blood metabolites were used as exposure factors and genome-wide association studies of RA as endpoints, and MR analysis was used to analyze the relationship between the 2. Inverse-variance weighting (IVW) was the preferred method, and 4 methods, MR-Egger method, weighted median method, simple mode method and weighted mode method, were used as supplements to the analysis process. Cochran *Q* test, MR-Egger intercept test, MR-PRESSO, and leave-one-out method analysis were also selected for sensitivity analysis. The validity of the results was verified by Bonferroni correction (*P* < .0357 × 10^−3^) and false discovery rate (FDR) test. In addition, reverse causality was excluded by Steiger test. A total of 77 metabolites were analyzed by MR to obtain a causal relationship with RA. Forty-seven known metabolites, 20 metabolite ratios, and 10 unknown metabolites were involved. Under the strict screening of Bonferroni correction and FDR test, docosatrienoate (22:3n3) levels i.e. docosatrienoic acid (DTA; *P*_IVW_ = 1.63 × 10^−5^, OR = 0.8859, CI = 0.8384–0.9361, *P*_FDR_ = .0229) was significantly associated with a reduced risk of RA. The results of the Cochran *Q* test (MR-Egger, Q’s = 14.5184, *P* = .8463; IVW.Q = 14.8882, *P* = .8670) and the MR-Egger regression intercept term of −0.0042, se = 0.0070, *P* = .5496 suggested that the absence of heterogeneity (*P* > .05) and horizontal pleiotropy (*P* > .05) among single nucleotide polymorphisms, the funnel plot and MR-PRESSO method further demonstrated the robustness of the results. In addition, Steiger test (*P* = 3.0752 × 10^−138^) excluded the presence of reverse causality. The present study provides evidence for the existence of a causal relationship between 77 especially DTA and RA by screening 1400 human blood metabolites. This study helps to reveal the biological mechanisms of RA and provides new ideas for the prevention and treatment of RA.

## 1. Introduction

Rheumatoid arthritis (RA) is an autoimmune disease with a chronic inflammatory response. RA results in joint hyperplasia and inflammation, causing irreversible damage to multiple joints, leading to disability and even death.^[1–3]^ The prevalence of RA ranges from 0.4% to 1.3%, with a higher incidence in females, which is 2 to 3 times that in males, and the incidence increases with age. The prevalence increases with age, with the highest prevalence in the 50 to 59 years of age.^[4,5]^ A 2008 study on the prevalence of RA indicated that more than 1.3 million patients in the United States suffer from the disease, which is a heavy burden.^[6,7]^

Metabolomics, as an important element of biology, based on genomics, proteomics, etc, provides new ideas to explore the mechanism and prevention and control of diseases. Metabolites are small molecules that are intermediate or final products of human metabolic processes and are important for in-depth study of diseases. A scientific study in 2022 established relevant disease loci for metabolites and proposed a mechanism for correlating traits between diseases.^[8]^ This has important implications for us to explore the occurrence and treatment of diseases through metabolism.

RA has been explored as a complex metabolic dysfunction disease in previous studies, for example, Lingxia Xu et al in a 2022 review furthered our understanding of RA by illustrating that methotrexate drugs corrected triglyceride and fatty acid levels through metabolomics.^[9]^ Existing studies have proved that metabolomics plays a very important role in the exploration of the disease, but currently there is not a rigorous data exploration of the relationship between metabolites and RA, and traditional studies inevitably have certain shortcomings, for example, Agnihotri P et al in a review study in 2021, illustrated the current status and progress of the metabolomics of RA, but due to the lack of a large amount of data to support, it is not possible to comprehensively analyze the metabolites associated with RA.^[10]^ For this reason, further investigation of the causal relationship between metabolites and RA became the main direction of this study.

Mendelian Randomization (MR) is a method that takes genetic variants as instrumental variables (IVs) to study the causal relationship between exposure factors and outcomes, in which single nucleotide polymorphisms (SNPs) are the most common genetic variants, which are innately acquired factors, which are usually not associated with the rest of the variables. Since genetics belongs to the innate existence and the disease occurs later, the order of innate and acquired variants cannot be reversed, so MR can exclude the interference of reverse causality,^[11]^ and at the same time, MR can also reduce the influence of confounding factors to a certain extent. Gu et al applied MR to explore the relationship between metabolites and osteoarthritis^[12]^; and Huang et al are also using MR analysis to explore the potential association of lipids and their modifiers with RA.^[13]^ There are more and more cases proving the feasibility of MR. However, there is still a lack of relevant studies in this field. The aim of this study was to further analyze the risk relationship between 1400 blood metabolites and RA by MR based on the genome-wide association studies (GWAS) database and to explore the metabolic processes related to RA.

## 2. Materials and methods

### 2.1. Study design

**Figure 1.**
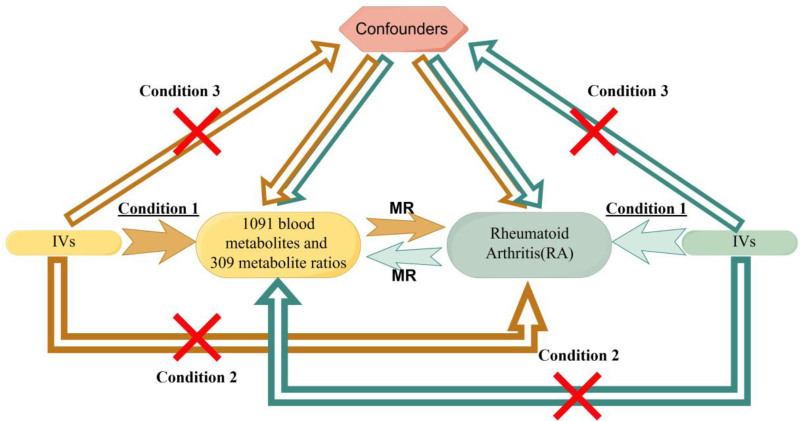
Three conditions for MR analysis. MR = Mendelian randomization, RA = rheumatoid arthritis.

This study investigates the relationship between 1400 blood metabolites and the risk of RA by MR, with metabolites as the exposure factors and RA as the outcome. The whole study process should fulfill 3 basic conditions: The selected metabolite SNPs should be strongly correlated with exposure factors, which meets the association hypothesis; the selected metabolite SNPs are not strongly correlated with the outcome RA, which excludes the exclusion hypothesis; and the selected metabolite SNPs can’t influence the outcome by influencing other factors related to the outcome RA, which excludes the independence hypothesis (Fig. 1).

### 2.2. Data sources

#### 2.2.1. Data sources for metabolites

The human blood-related metabolite database was obtained from the Genomics GWAS database (http://metabolomics.helmholtz-muenchen.de/gwas/). The metabolite data used in this study came from a metabolite screen conducted by Chen et al^[14]^ in 2023 based on GWAS, which is noteworthy for being the most comprehensive metabolite screen to date, in detail, based on 8299 individuals from the Canadian longitudinal study of aging containing 1091 metabolites and 309 metabolite ratios totaling 1400. Known metabolites can be attributed to the broad metabolite groups included in the Kyoto encyclopedia of genes and genomes database (carbohydrate, energy, lipid, nucleotide, amino acid, xenobiotics, peptides, cofactors and vitamins).

#### 2.2.2. Data sources for RA

The GWAS for RA was obtained from the FinnGen database (https://www.finngen.fi/en) with the search formula finngen_R10_M13_RHEUMA, which included 13,621 RA patients and 262,844 controls, and all RA case diagnoses were referenced to the International Classification of Diseases, 10th edition (ICD-10) in the M13 codes. The FinnGen research project integrates genetic data on disease endpoints from the Finnish Biobank and aims to determine genotype-phenotype correlations in the Finnish population.

### 2.3. Selection of instrumental variables

The parameters *P* < 5 × 10^−6^, *r*^2^ < 0.01, and kb = 10,000 were set to screen SNPs for metabolites and RA based on the selection conditions, extract data on exposure factors, remove chain imbalances, and perform dichotomous variables to calculate ORs (ORs > 1 for risk factors and vice versa for protective factors). The setting was set to screen IVs with *F*-statistic values >10 to minimize bias in the results caused by weak IVs.^[15]^ The resulting SNPs were brought into the PhenScanner database (http://www.phenoscanner.medschl.cam.ac.uk/) for validation to exclude SNPs directly related to outcome.

### 2.4. Mendelian randomization analysis

Five different MR analysis methods were used in this study to explore the risk relationship between metabolites and RA: the inverse-variance weighted (IVW) method, the MR-Egger method, the Weighted median method, the Simple mode method, and the Weighted mode method. It is worth mentioning that the IVW method, which uses all valid SNPs, is the most frequently used and effective computational method in MR analysis, and IVW can provide the most accurate and effective causal estimation,^[16,17]^ so our study uses the IVW method as the main research method. Of course, we still used the other 4 methods as an adjunct to further validate the relationship between metabolites and the risk of RA.

### 2.5. Statistical analysis

False discovery rate (FDR) correction was used to control for false positives and improve the reliability of conclusions in multiple testing. Statistically significant associations were considered if the FDR of the estimated causal effect for the metabolite was < .05. In addition to this, we used the Bonferroni correction, a multiple test correction, to corroborate the strong validity of the data. The corrected significance threshold was set at 0.05/1400 (metabolite species) = 0.0357 × 10^−3^, and the results were considered suggestive when 0.0357 × 10^−3^ ≤ *P* < .05, and the causal effect between exposure and outcome was considered significant when *P* < .0357 × 10^−3^. Such statistical results further validate the scientific validity and effectiveness of our study.

### 2.6. Sensitivity analysis

The IVW method, although used as the main method in MR analysis, cannot effectively exclude the effects of horizontal pleiotropy and heterogeneity. Heterogeneity represents the variability of causal estimates of SNPs, and the lower the heterogeneity, the more reliable the results of MR.^[18]^ We adopted a dual approach of Cochran *Q* and funnel plot to test the heterogeneity of the MR analysis of the current metabolites with RA.^[19]^ MR of multiple residuals and outliers (Pleiotropy residual sum and outlier, MR-PRESSO) and MR-Egger regression intercept method were used as effective methods to validate horizontal pleiotropy.^[20]^ In the presence of outliers, causality should be re-estimated after removing the outliers. In addition, we used leave-one-out sensitivity analysis, which means that after excluding the SNPs of each metabolite, the unexcluded SNPs did not affect the results, to determine the effect of each SNP on the results of MR analysis. All MR analyses and tests were conducted using the “TwoSampleMR” and “MRPRESSO” packages in R software (version 4.3.1).

## 3. Results

### 3.1. Selection of instrumental variables

After screening 1400 blood metabolite IVs, a median IV of 23 SNPs was derived, ranging from 11 to 85 SNPs (3-hydroxylaurate levels were the least and X-15523 levels were the most). Six SNPs with *F* <10 (rs72629869, rs112957900, rs72728613, rs114453281, rs539062096, rs74967281) were excluded, and the statistical values of *F* for the remaining SNPs were all >10 (with a minimum value of 10.0248), suggesting that a weak instrumental bias is unlikely to affect causal effect.

### 3.2. Results of the analysis

**Figure 2.**
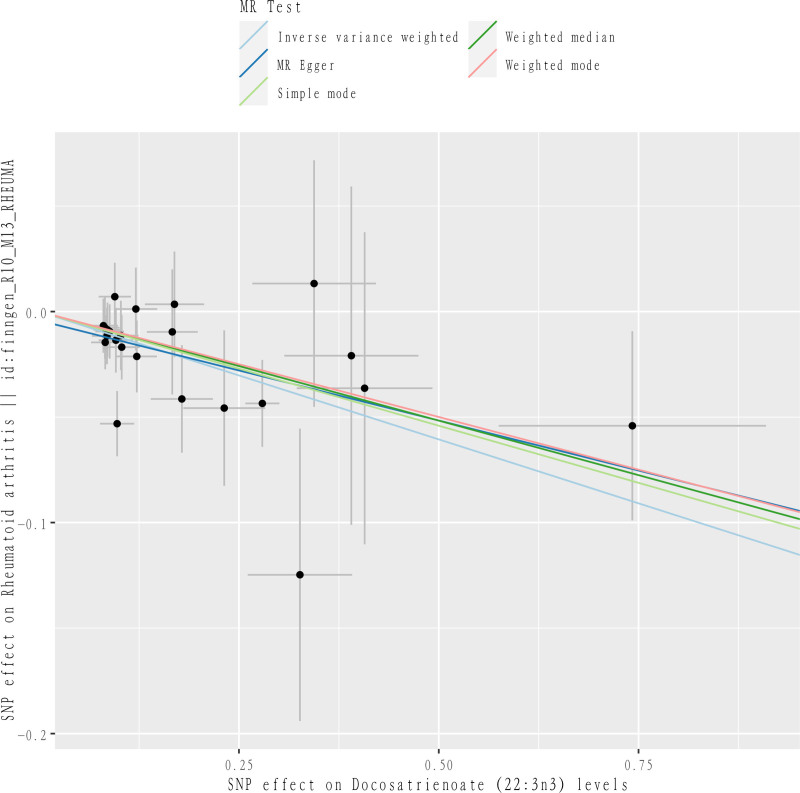
Scatter plot of the relationship between Docosatrienoate (22:3n3) and RA. RA = rheumatoid arthritis.

**Figure 3.**
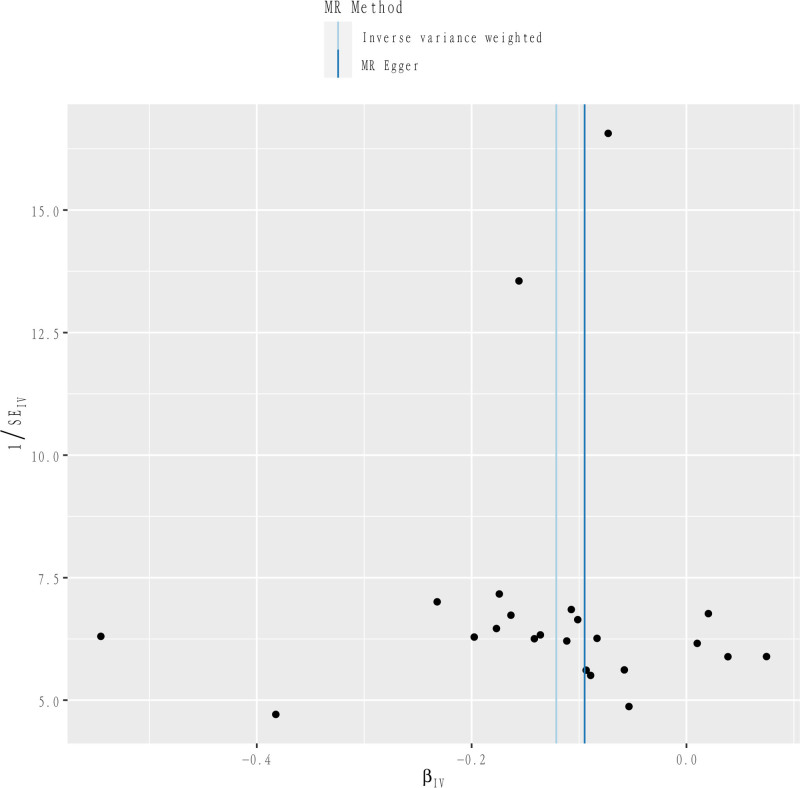
Funnel plot of the relationship between Docosatrienoate (22:3n3) and RA. RA = rheumatoid arthritis.

**Figure 4.**
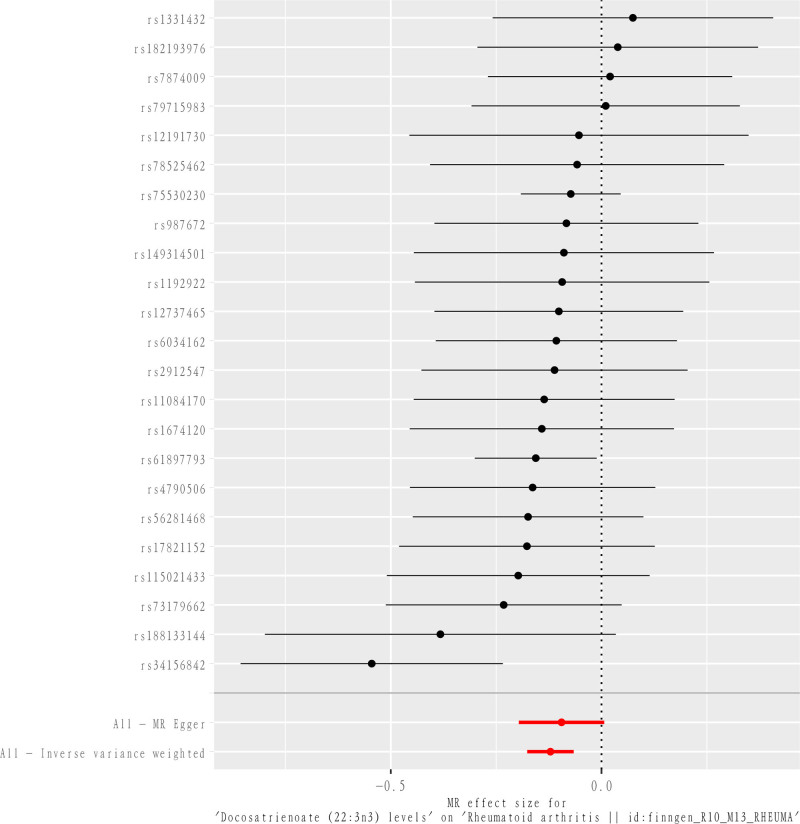
Forest diagram of the relationship between Docosatrienoate (22:3n3) and RA. RA = rheumatoid arthritis.

**Figure 5.**
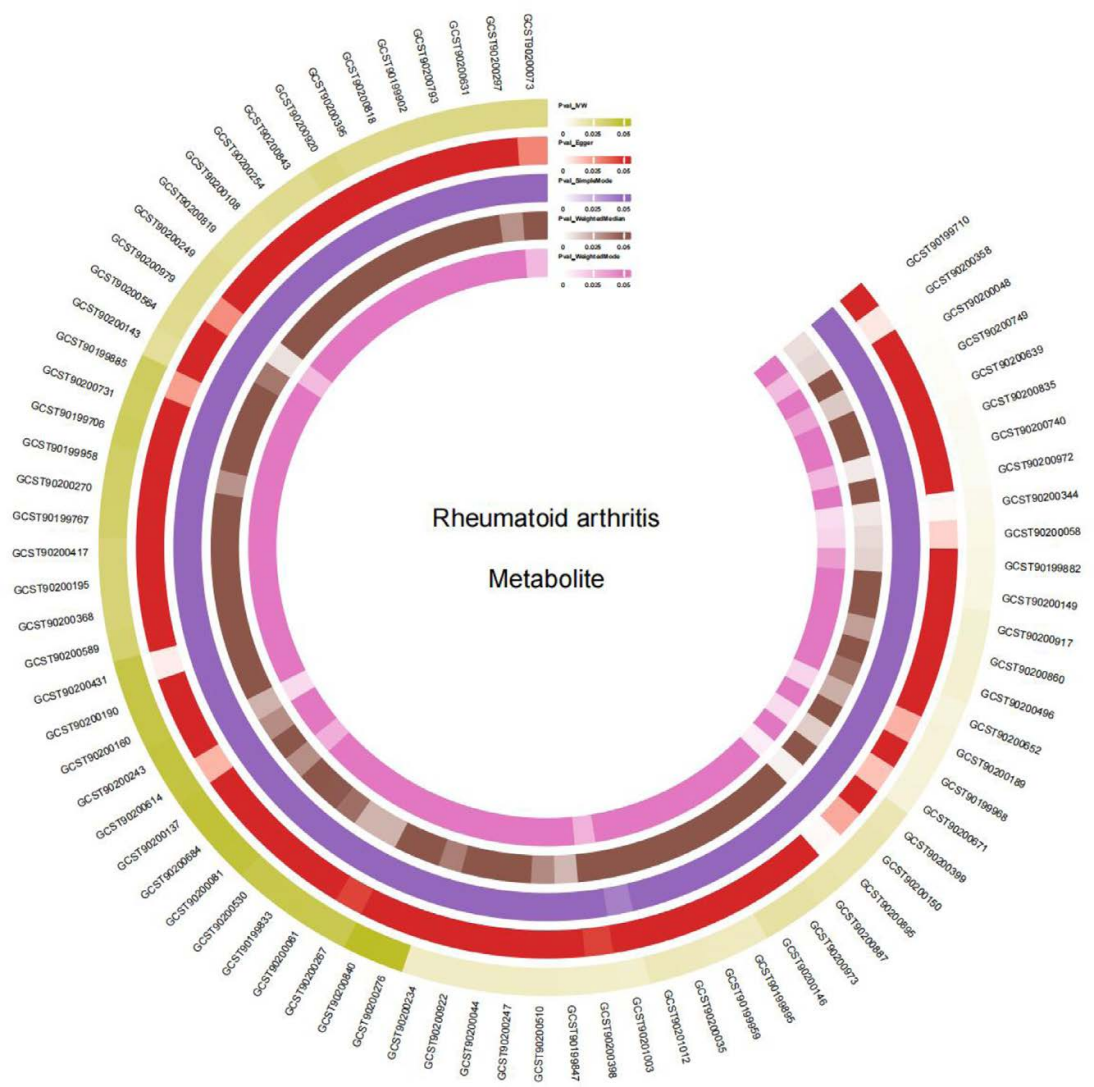
Circular heat map. The circle represents 5 methods (the inverse-variance weighted method; the MR-Egger method; the weighted media method; the simple mode method, and the weighted mode method).and the color represents the size of the *P*-value. MR = Mendelian randomization.

**Figure 6.**
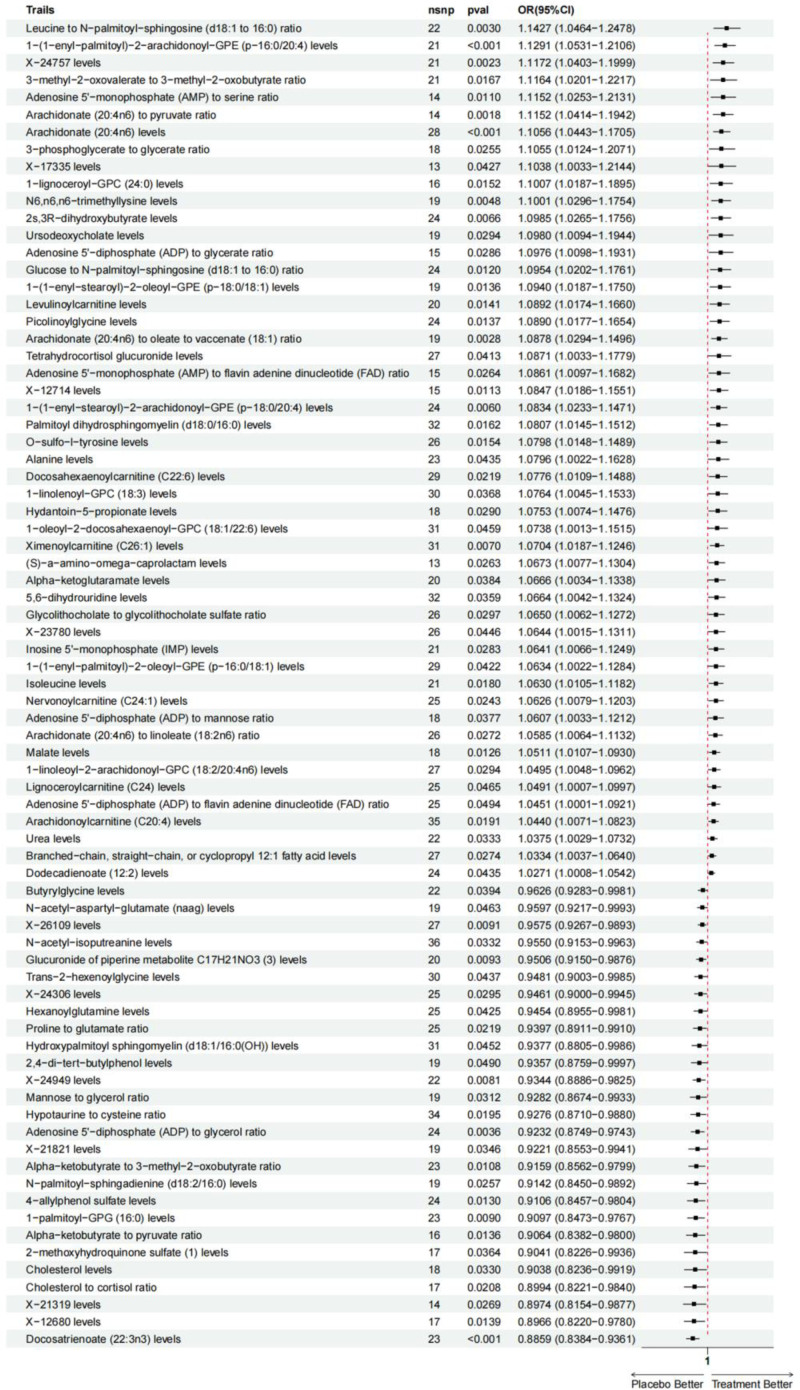
Composite forest plot of IVW methods for positive results in MR analysis. IVW = inverse-variance weighted, MR = Mendelian randomization, SNPs = single nucleotide polymorphisms.

After our MR analysis of metabolite-RA relationships, we found that a total of 77 out of 1091 metabolites and 309 metabolite ratios had interrelated causal relationships with RA. The most significant association between docosatrienoate (22:3n3) levels and RA was found to be the most significant (*P*_IVW_ = 1.63 × 10^−5^, OR = 0.8859, CI = 0.8384–0.9361, *P*_FDR_ = .0229, Table 1 and Figs. 2–4). The 23 SNPs at docosatrienoate (22:3n3) levels were brought into the PhenoScanner database, and it was found that there were no SNPs directly associated with outcome RA, and the statistics of docosatrienoate (22:3n3) levels were consistent with the setting of *P*_IVW_ < 0.0357 × 10^−3^ and FDR < 0.05. In addition, in the MR-Egger method (*P* = .0807, OR = 0.9096, CI = 0.8220–1.0065), Weighted median method (*P* = .0090, OR = 0.9018, CI = 0.8345–0.9745), Simple mode method (*P* = .1181, OR = 0.8974, CI = 0.7877–1.0224), weighted mode method (*P* = .0663, OR = 0.9049, CI = 0.8177–1.0014) (see table for details) in which the MR-Egger and Weighted median methods and IVW methods are highly consistent, while the Simple mode method and Weighted mode method were not statistically significant compared to the results of the IVW method. As 2 different estimation methods in MR analysis, Weighted mode method is based on the variance of genetic tools, while Simple mode method does not take into account the effect of variance, so Simple mode method produces relatively inaccurate analysis results. And the reason for the lack of statistical significance of Weighted mode method may lie in the bias of genetic tools.^[21]^ Our study was mainly analyzed by IVW method, which provided results with the highest validity among the 5 methods. So the results of simple mode method and weighted mode method which were not statistically significant were determined by our study to be invalid and do not have an impact on the final analytical argument. Taken together, the results of the analysis demonstrated that docosatrienoate (22:3n3) levels were strongly causally associated with RA and were protective factors against RA. In addition to this, a total of 76 metabolites were suggestively associated with RA (Figs. 5 and 6). Forty-nine of them were positively associated with the risk of RA, including 32 known metabolites such as 1-(1-enyl-palmitoyl)-2-arachidonoyl-GPE (*P*-16:0/20:4) levels, etc, and also including 13 metabolite ratios and 4 unknown metabolites; 27 of them were associated with a reduced risk of RA, including 15 known metabolites such as 1-palmitoyl-GPG (16:0) levels, etc, as well as 7 metabolite ratios and 6 unknown metabolites.

**Table 1:** Detailed results of docosatrienoate (22:3n3) levels.

Explore	Method	nsnp	*P*-value	OR	or_lcl95	or_ucl95
Docosatrienoate (22:3n3) levels (docosatrienoic acid, DTA)	IVW	23	1.63×10^−5^	0.8859	0.8384	0.9361
	MR-Egger		.0807	0.9096	0.8220	1.0065
	Weighted median		.0090	0.9018	0.8345	0.9745
	Simple mode		.1181	0.8974	0.7877	1.0224
	Weighted mode		.0663	0.9049	0.8177	1.0014

DTA = docosatrienoic acid, IVW = inverse-variance weighting, MR = Mendelian randomization, OR = odds ratio.

### 3.3. Sensitivity analysis

**Figure 7.**
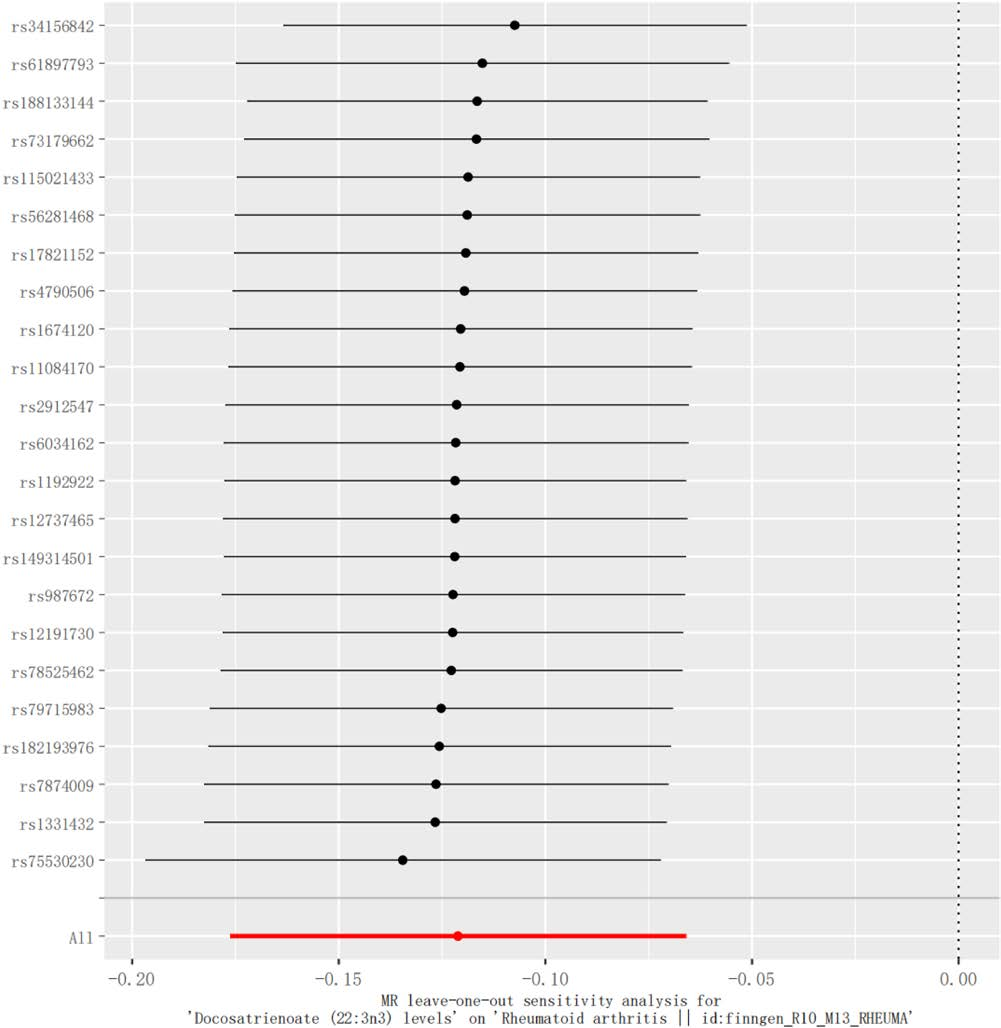
Mendelian randomization sensitivity analysis of the relationship between Docosatrienoate (22:3n3) and RA using the retention analysis method. RA = rheumatoid arthritis.

Although IVW’s method is sufficiently valid, in order to prevent bias caused by IVs, our study will further assess its heterogeneity and horizontal multiplicity to confirm the robustness of the results. In assessing the heterogeneity, the statistic of *Q* for MR-Egger result of Cochran *Q* test was 14.5184, *P* = .8463, and the statistic of *Q* for IVW was 14.8882, *P* = .8670, and the funnel plot also corroborated that the results had a small degree of heterogeneity (Fig. 7). The MR-Egger regression intercept term in the test for horizontal multiplicity was −0.0042, se = 0.0070, *P* = .5496, while the MR-PRESSO results also indicated no horizontal multiplicity (*P* = 2.959255 × 10^−5^). The funnel plot representation of the leave-one-out sensitivity analysis did not indicate heterogeneity or horizontal pleiotropy. In addition, we added the Steiger test to determine the directionality of the causal relationship between metabolites and RA, and the *P*-value for docosatrienoate (22:3n3) levels = 3.0752 × 10^−138^, which is a very small *P*-value (*P* < .001) indicating that SNPs correlate more with metabolites than with RA correlation with metabolites, and no reverse causality would occur.

### 3.4. Relevant metabolic pathways

In order to explore the causal relationship between metabolites and RA more deeply, we enumerated the metabolic pathways of the known metabolites in the positive results, which were used to analyze the relationship between RA and metabolites through the aspect of metabolic pathways (Tables 2 and 3).

**Table 2:** Known metabolites and their main metabolic pathways associated with reduced RA risk.

Metabolites	SUPER_PATHWAY	SUB_PATHWAY	HMDB
Docosatrienoate (22:3n3)	Lipid	Long chain polyunsaturated fatty acid (n-3 and n-6)	HMDB0002823
1-Palmitoyl-GPC (16:0)	Lipid	Lysophospholipid	HMDB0010382
4-Allylphenol sulfate	Xenobiotics	Food component/plant	–
N-Palmitoyl-sphingadienine (d18:2/16:0)	Lipid	Ceramides	–
Glucuronide of piperine metabolite C17H21NO3 (3)	Xenobiotics	Food component/plant	–
Cholesterol	Lipid	Sterol	HMDB0000067
N-acetyl-isoputreanine	Amino Acid	Polyamine metabolism	HMDB0094713
2-Methoxyhydroquinone sulfate (1)	Xenobiotics	Benzoate metabolism	–
Butyrylglycine	Lipid	Fatty acid metabolism (also BCAA metabolism)	–
Hexanoylglutamine	Lipid	Fatty acid metabolism (acyl glutamine)	–
Hydroxypalmitoyl sphingomyelin (d18:1/16:0 (OH))	Lipid	Sphingomyelins	–
NAAG	Amino acid	Glutamate metabolism	HMDB0001067
Trans-2-hexenoylglycine	Lipid	Fatty acid metabolism (acyl glycine)	–
Lignoceroylcarnitine (C24)	Lipid	Fatty acid metabolism (acyl carnitine, long chain saturated)	HMDB0240665
2,4-di-tert-butylphenol	Xenobiotics	Chemical	HMDB0013816

NAAG = N-acetyl-aspartyl-glutamate, RA = Rheumatoid arthritis Rheumatoid arthritis.

**Table 3:** Known metabolites and their main metabolic pathways associated with increased RA risk.

Metabolites	SUPER_PATHWAY	SUB_PATHWAY	HMDB
Aachidonate (20:4n6)	Lipid	Long chain polyunsaturated fatty acid (n-3 and n-6)	HMDB0001043
Inosine 5’-monophosphate (IMP)	Nucleotide	Purine metabolism, (hypo)xanthine/inosine containing	HMDB0000175
Isoleucine	Amino acid	Leucine, isoleucine and valine metabolism	HMDB0000172
Ursodeoxycholate	Lipid	Secondary bile acid metabolism	HMDB0000946
2S,3R-dihydroxybutyrate	Lipid	Fatty acid, dihydroxy	HMDB0002453
Hydantoin-5-propionate	Amino acid	Histidine metabolism	HMDB0001212
1-Lignoceroyl-GPC (24:0)	Lipid	Lysophospholipid	HMDB0010405
O-sulfo-L-tyrosine	Xenobiotics	Chemical	HMDB0155722
1-(1-Enyl-stearoyl)-2-oleoyl-GPE (*P*-18:0/18:1)	Lipid	Plasmalogen	HMDB0011375
Palmitoyl dihydrosphingomyelin (d18:0/16:0)	Lipid	Dihydrosphingomyelins	HMDB0010168
1-(1-Enyl-stearoyl)-2-arachidonoyl-GPE (*P*-18:0/20:4)	Lipid	Plasmalogen	HMDB0005779
1-(1-Enyl-palmitoyl)-2-arachidonoyl-GPE (*P*-16:0/20:4)	Lipid	Plasmalogen	HMDB0011352
1-Linoleoyl-2-arachidonoyl-GPC (18:2/20:4n6)	Lipid	Phosphatidylcholine (PC)	HMDB0008147
1-(1-Enyl-palmitoyl)-2-oleoyl-GPE (*P*-16:0/18:1)	Lipid	Plasmalogen	HMDB0011342
1-Oleoyl-2-docosahexaenoyl-GPC (18:1/22:6)	Lipid	Phosphatidylcholine (PC)	HMDB0008123
Ximenoylcarnitine (C26:1)	Lipid	Fatty acid metabolism (acyl carnitine, monounsaturated)	–
Arachidonoylcarnitine (C20:4)	Lipid	Fatty acid metabolism (acyl carnitine, polyunsaturated)	HMDB0006455
Docosahexaenoylcarnitine (C22:6)	Lipid	Fatty acid metabolism (acyl carnitine, polyunsaturated)	–
Nervonoylcarnitine (C24:1)	Lipid	Fatty acid metabolism (acyl carnitine, monounsaturated)	–
Hydroxy-N-6,N-6,N-6-trimethyllysine	Amino Acid	Lysine metabolism	–
Levulinoylcarnitine	Xenobiotics	Food component/plant	–
Picolinoylglycine	Lipid	Fatty acid metabolism (acyl glycine)	HMDB0059766
(S)-a-amino-omega-caprolactam	Xenobiotics	Food component/plant	–
Branched-chain, straight-chain, or cyclopropyl 12:1 fatty acid	Partially Characterized Molecules	Partially characterized molecules	–
Malate	Energy	TCA cycle	HMDB0031518
Urea	Amino Acid	Urea cycle; arginine and proline metabolism	HMDB0000294
5,6-dihydrouridine	Nucleotide	Pyrimidine metabolism, uracil containing	HMDB0000497
1-linolenoyl-GPC (18:3)	Lipid	Lysophospholipid	HMDB0010388
Alpha-ketoglutaramate	Amino acid	Glutamate metabolism	HMDB0001552
Tetrahydrocortisol glucuronide	Lipid	Corticosteroids	–
Dodecadienoate (12:2)	Lipid	Fatty acid, dicarboxylate	–
Alanine	Amino acid	Alanine and aspartate metabolism	HMDB0000161

## 4. Discussion

In our present MR analysis, a total of 77 metabolites were found to be causally associated with RA, involving 6 metabolite species such as lipid, amino acid, etc, among which, the Bonferroni-corrected *P*-value showed a significant causal association between docosatrienoate (22:3n3) levels and reduced genetic risk of RA, and sensitivity analyses further confirmed the robustness of the results. With the results of the present analysis, we obtained docosatrienoate (22:3n3) as a metabolite with protective factors against RA, which we can use to guide our clinical work by studying it at a deeper level. Notably, the current study is the first MR analysis experiment to explore the risk relationship with RA using 1400 relevant metabolites. Our study provides a new direction for gene-metabolism-disease and new insights into the prevention and treatment of RA in the future.

RA has long been a heavy burden on human health,^[22]^ which has created an urgent need for early screening and prevention of RA. Metabolomics is an excellent tool that greatly aids in the in-depth clinical study of the disease. According to statistics, the pathogenesis of RA is mainly genetic,^[23]^ which accounts for about 50% of the total incidence. RA, as an inflammatory arthritis, has an extremely complex process of cartilage destruction and inflammation formation. First of all, the synovial membrane consists of fibroblasts and macrophages called synoviocytes, and the synovial membrane is only a very thin layer in ordinary people, but in patients with RA, due to the abnormal proliferation of synoviocytes, a thicker structure known as “cataracts” is formed, and the synovial fibroblasts are highly destructive at this time. In addition, inflammation in the joints is greatly related to the production of tumor necrosis factor (TNF) and interleukin (IL) due to the imbalance between B cells, T cells, macrophages, etc.^[24–26]^

Docosatrienoate (22:3n3) levels, also known as docosatrienoic acid (DTA), is 1 of the n-3 very long chain polyunsaturated fatty acids (VLCPUFAs), which are an important part of cell membranes and precursors of metabolized biocompounds in humans.^[27]^ Fluctuating levels may contribute to neurological or immune system disorders.^[28]^ The main metabolic pathway of DTA is Long Chain Polyunsaturated Fatty Acid (n-3 and n-6). In RA, n-6 represents the pro-inflammatory effect, while n-3 mainly exerts its anti-inflammatory effect. n-3 fatty acids are mainly derived from the conversion of n-3 Faα-linolenic acid (ALA), which is seldom synthesized endogenously in humans, and is mainly consumed through dietary intake,^[29]^ and therefore, it is mainly extracted from fatty marine products, such as fish oils. n-3 pathway is the main source of DTA, and ALA is extended into eicosatriene. ALA is extended to eicosatrienoic acid (ETA, 20:3n-3), which is catalyzed by a single ELO-type elongase (EhELO1) to become DTA; similarly, DTA is produced from eicosatrienoic acid (EDA, 20:2n-6) in the n-6 pathway, which is subsequently catalyzed by VLCPUFA ω3 desaturase (PiO3) to produce DTA from ETA.^[27]^ Previous studies have shown that n-3 polyunsaturated fatty acids especially eicosapentaenoic acid, and docosahexaenoic acid (DHA) have been shown to be potent metabolites beneficial for RA symptom improvement. The intake of polyunsaturated fatty acids can effectively counteract the cellular levels of n-6 fatty acids, especially arachidonic acid AA, which inhibits the AA-produced inflammatory factors to infiltrate and damage RA, in addition to the production of cartilage-explaining proteases during metabolism to reduce the migration of leukocytes, and other channels to produce a positive effect on RA.^[30–33]^ In an experimental study conducted by Yi et al in 2021, DTA was found to be effective in reducing the expression of the pro-inflammatory factors interleukin-1b (IL-1b), interleukin-6 (IL-6), and TNF-a, which, along with other representative n-3 polyunsaturated fatty acids, also have powerful anti-inflammatory effects.^[34]^ DTA itself belongs to n-3 unsaturated fatty acids along with DHA, which may be related to the fact that n-3 unsaturated fatty acids have the ability to regulate the expression of anti-inflammatory factors to achieve anti-inflammatory effects. However, the current study mainly focuses on eicosapentaenoic acid and DHA, ignoring the effects of other n-3 unsaturated fatty acids. Therefore, from the results of the present MR analysis and a survey of the current studies, we believe that the levels of docosatrienoate (22:3n3) could prove to be a metabolite associated with a reduced risk of RA.

In addition, we identified 76 metabolites associated with RA, which have also appeared in previous studies, such as the well-known “lipid paradox,”^[35]^ which explores the relationship between cholesterol and RA, and is a confirmation of the risk association between cholesterol levels and RA in our positive results. This is also a confirmation of the risk association between cholesterol levels and RA in our positive results. Or the previously mentioned pro-inflammatory effect of arachidonic acid on RA was confirmed in the analyzed results. In addition, we found that the metabolites in the results of the current MR study were predominantly lipids, accounting for about 56% of all known metabolites. Lei et al systematically outlined the effects of lipids on RA immune cells, macrophages, dendritic cells, and so on, in a review study in 2023^[36]^; and another study in 2015 also analyzed the lipid metabolism associated with RA in a more complete manner.^[37]^ In addition, fatty acid metabolism is also greatly represented by the enumeration of common metabolic pathways of positive metabolites. From this, it can be inferred that lipid metabolism has a great weight in the occurrence and progression of RA, which is consistent with the results of our analysis. Meanwhile, we also found many other known metabolites, unknown metabolites and metabolite ratios causally related to RA, but with the current study can’t prove the specific link between them and RA, and still need to be explored in further research.

There are many advantages of this MR analysis, such as our first MR analysis of RA using data related to 1400 serum metabolites, which covers the largest currently known genetic variables and involves data on 1400 related metabolites, covering 13,621 individuals with RA. These 2 GWAS databases allowed us to do a relatively systematic study of the relationship between metabolites and RA risk. In addition to that. We applied sensitivity analysis and Steiger test to eliminate the interference caused by confounding factors and to determine the accuracy of our conclusions, so the results of this study on the relationship between metabolites and RA risk were recognized as scientific and valid by us.

Of course, there are some limitations in this study. First, the metabolite data came from blood extraction, which is considered an excellent source of data, but the metabolic process could not be analyzed completely due to the fact that RA has data on joint cavity fluid and synovial tissues specific to RA in addition to blood. Second, some of the metabolites in our results have not been explored in studies related to RA, which is a limitation to the elaboration of the results of our current MR analysis. Finally, the data of our RA came from a database of the Finnish population, which may ignore the findings of other races and do not allow for multiracial inferences. Therefore, we hope to conduct a more extensive analysis in the future to further explore the metabolic processes and related targets of RA.

## 5. Conclusion

In summary, this is the first time to our knowledge that we have used a genome-wide database to perform MR analysis of risk associations between metabolites and RA. We identified 77 metabolites that were causally associated with RA and identified docosatrienoate (22:3n3) levels, an n-3 fatty acid that was strongly associated with reduced risk of RA. In conclusion, our present study provides highly valuable ideas on metabolites as potential therapeutic targets for the disease, as well as new insights into the metabolic analysis of RA.

## Acknowledgments

All authors greatly appreciate the reviewer’s editing of the manuscript.

## Author contributions

**Conceptualization:** Hao Liu.

**Data curation:** DeZhi Yan, Hao Liu.

**Funding acquisition:** Di Luo.

**Investigation:** Di Luo.

**Methodology:** Wei Yan, JinSong Li.

**Software:** DeZhi Yan.

**Supervision:** Wei Yan.

**Visualization:** JinSong Li.

**Writing – original draft:** DeZhi Yan.

**Writing – review & editing:** Di Luo, Wei Yan, JinSong Li.
